# Educational and Ethical Considerations for Genetic Test Implementation Within Health Care Systems

**DOI:** 10.1089/nsm.2019.0010

**Published:** 2020-05-26

**Authors:** Emma Kurnat-Thoma

**Affiliations:** ^1^ Department of Intramural Research, DHHS/NIH/NINR, Bethesda, Maryland, USA.; ^2^ School of Nursing and Health Studies, Georgetown University, Washington, District of Columbia, USA.

**Keywords:** ethical legal social implications, genetic tests, health care provider training and education, health care system implementation

## Abstract

**Introduction:** The precision medicine (PM) era presents unprecedented proliferation of genetic/genomic initiatives, information, and bioinformatic tools to enhance targeted molecular diagnosis and therapeutic treatments. As of February 29, 2020, the National Institutes of Health (NIH) National Center for Biotechnology Information (NCBI) Genetic Testing Registry contained 64,860 genetic tests for 12,268 conditions and 18,686 genes from 560 laboratories, and the Food and Drug Administration had 404 entries for pharmacogeneomic biomarkers used in drug labeling. Population-based research initiatives including NIH's *All of Us* and Veterans Affairs' *Million Veteran Program*, and the UK Biobank, combine use of genomic biorepositories with electronic medical records (i.e., National Human Genome Research Institute's [NHGRI's] electronic Medical Records and Genomics [eMERGE] Network). Learning health care systems are implementing clinical genomics screening programs and precision oncology programs. However, there are insufficient medical geneticists, nurse geneticists, and genetics counselors to implement expanding number of clinical genetic tests that are required for PM implementation.

**Methods:** A scoping review of current (2014–2019) trends in U.S. genomic medicine translation, PM health care provider workforce education and training resources, and genomic clinical decision support (CDS) implementation tools was conducted.

**Results:** Health care delivery institutions and systems are beginning to implement genetic tests that are driving PM, particularly in the areas of oncology, pharmacogenetics, obstetrics, and prenatal diagnostics. To ensure safe adoption and clinical translation of PM, health care systems have an ethical responsibility to ensure their providers and front-line staff are adequately prepared to order, use, and interpret genetic test information.

**Conclusion:** There are a number of high-quality evidenced-based educational resources and CDS tools available. Strong partnerships between health care system leaders, front-line providers and staff coupled with reasonable goal setting can help drive PM translation interests.

## Introduction

Precision medicine (PM) is an approach to treatment and prevention of disease that takes into account a patient's genes, lifestyle, and environmental characteristics.^1^ The PM era presents unprecedented proliferation of genetic/genomic information and bioinformatic tools to enhance molecular diagnosis and therapeutic treatments. There has been rapid growth in both number of genetic tests and Food and Drug Administration (FDA) approvals for drugs with pharmacogenetic labeling information. As of February 29, 2020, the National Institutes of Health (NIH) National Center for Biotechnology Information (NCBI) Genetic Testing Registry contained 64,860 genetic tests for 12,268 conditions and 18,686 genes from 560 laboratories, and the FDA had 404 entries for pharmacogenomic biomarkers used in drug labeling.^2,3^

Population-based biobank research initiatives including NIH's *All of Us*, Veterans Affairs' *Million Veteran Program*, and the UK Biobank combine use of genomic biorepositories with electronic medical records (i.e., National Human Genome Research Institute's [NHGRI] electronic Medical Records and Genomics [eMERGE] Network) to gain novel insights into candidate genes targets and complex gene–lifestyle interactions fueling development of chronic multifactorial diseases.^4–6^ Learning health systems are beginning to implement clinical genomics screening programs to identify and reduce patient risk for development of adverse outcomes and diseases such as familial hypercholesterolemia and hereditary cancer syndromes, and PM-based treatments for oncology community health care settings.^7,8^

Although these large-scale research initiatives began the PM movement, the field is rapidly evolving and broadening beyond genetics/genomics to include information generated from wearable sensors and digital health devices, network biology and systems medicine, polygenic risk score prediction algorithms, bioinformatics data science and machine learning methodologies, and multiomics profiling for a variety of health and disease states (Table 1).^9–15^

**Table 1.** Current and Emerging Terminology, Paradigms in Genomic Health Care

Term	Definition
Genomic medicine	“Emerging medical discipline that involves using genomic information about an individual as part of their clinical care (e.g., for diagnostic or therapeutic decision-making) and the health outcomes and policy implications of that clinical use.”^16^
Network medicine	“Emerging field that combines systems biology and network science. It runs counter to the prevailing scientific reductionist trend that dominates current medical research on disease etiology and treatment. Reductionism relies on single molecules or single genes to provide comprehensive and robust insights into the pathophysiology of complex diseases. Similarly, current drug development methodologies target single molecules that very frequently fail because of the unforeseen and unintended effects that result from the application of this piecemeal approach to pharmacology. Emphasizes a more holistic approach through the identification and investigation of networks of interacting molecular and cellular components. When network medicine is integrated into biomedical research, it has the potential to transform investigations of disease etiology, diagnosis, and treatment.”^11,17^
Personalized medicine	“Emerging practice of medicine that uses an individual's genetic profile to guide decisions made in regard to the prevention, diagnosis, and treatment of disease. Knowledge of a patient's genetic profile can help doctors select the proper medication or therapy and administer it using the proper dose or regimen.”^18^
Precision medicine	“An emerging approach for disease treatment and prevention that takes into account individual variability in genes, environment, and lifestyle for each person.”^1^ In cancer, precision medicine “uses specific information about a person's tumor to help diagnose, plan treatment, find out how well treatment is working, or make a prognosis.”^19^
Systems medicine	“New and emerging field that leverages complex computational tools to develop personalized assessments of disease risk and potential management options. Systems medicine seeks to enhance individualized diagnosis, prognosis, and treatment options. The introduction of Big Data has begun to require new types of physicians and biomedical scientists, which exploit modern computational sciences to process and analyze enormous quantities of information.”^9,10,20^
Precision health	“Precision health is broader—it includes precision medicine but also approaches that occur outside the setting of a doctor's office or hospital, such as disease prevention and health promotion activities.” This includes using targeted health information from patients' wearable sensors, implantable monitoring devices, and mobile devices for health prevention interventions.”^21^
Precision public health	“Improving the ability to prevent disease, promote health, and reduce health disparities in populations by applying emerging methods and technologies for measuring disease, pathogens, exposures, behaviors, and susceptibility in populations; and developing policies and targeted implementation programs to improve health.”^22^

Network medicine definition quote is verbatim from *Harvard Catalyst,* precluded by Chan and Loscalzo.^11^ Systems medicine definition quote is verbatim from *1st International Conference in Systems Network Medicine Conference,* precluded by Auffray et al.^9^ and Auffray.^10^

Artificial intelligence—the use of computing frameworks, algorithms, and theories to facilitate tasks that normally require human reasoning and decision making, understanding, or perception to include techniques such as machine learning and natural language processing—is emerging as a key analytic tool to manipulate and derive meaningful and actionable insights within the vast amounts of big data from whole genome sequence, multiomics data, electronic health records (EHRs), and others.^23^

Novel genetic/genomic/-omics insights gleaned from technological, engineering, and computer science approaches to identify disease biology mechanisms, predict onset, and implement targeted treatments are envisioned to shift health care from a reactive practice model to a proactive model.^9,10^ All health care paradigms utilizing genomic and molecular -omic data listed in Table 1 require the use of genetic tests, which are defined as “analysis of human DNA, RNA, chromosomes, proteins, and certain metabolites in order to detect heritable disease-related genotypes, mutations, phenotypes, or karyotypes for clinical purposes.”^24^

It is broadly recognized that genetics/genomics health care professionals are most readily able to use and to guide implementation of genetic tests into health care workflows; however, their numbers and scope are limited. Thus ensuring adequate workforce capacity and clinician education/training in these areas (for both genetics and nongenetics providers) is an ethical imperative to ensure clinical translation of PM across populations and within communities.

When the U.S. Congress appropriated funding for the Human Genome Project in 1990, a portion of its funding was devoted to the ethical, legal, and social implications (ELSI) of genetics/genomics research. In the light of any rapidly evolving biomedical scientific field, but particularly PM, there is an ethical duty to consider the implementation impacts of those technologies upon patients, their families, and society as a whole.^25^ The NHGRI ELSI portfolio has expanded since its initial funding and now spans multiple domains, including the implementation of genetic/genomic tests into health care settings and health systems so as to ensure fair access, use, and reimbursement.^26^

In accordance with these stated priorities, a scoping review was conducted with the following research question: “what are the current trends in U.S. genetic test use, genetics/genomics healthcare workforce education and training, and available clinical translation tools for healthcare systems?” Highlights from this article were presented as part of the “Ethics and Educational Considerations in the Era of Precision Medicine” panel on September 12, 2019, at the *1st International Conference in Systems and Network Medicine, Applications of Systems Science and Thinking to Biomedicine*.^20^

## Methods

A scoping review was performed to examine current trends (2014–2019) in U.S. health system genetic test use, health care provider workforce education and training in genetics/genomics, and available clinical decision support (CDS) tools for PM implementation. Biomedical and nursing databases searched included PubMed, CINAHL, and Google Scholar for international English articles of quantitative (clinical trials, implementation pilots, meta-analyses, and reviews), qualitative, and mixed-method research studies. MeSH search terminology criteria included Clinical Laboratory Improvement Amendments (CLIA)-certified laboratory genetic tests, health system implementation, genetic test implementation, ELSI, precision medicine/precision health genomic medicine implementation, workforce development, and CDS.

Specific health care disciplines reviewed included physicians, genetics counselors, and nurses, including gray literature on relevant credentialing and licensing statistics. Of the total 6242 records that were identified, 592 abstracts were reviewed, 186 full length reports and articles were read, and 51 full length articles were selected for the final scoping review. An additional 29 online resources for U.S. health system implementation of genetic tests were identified and practicable implementation frameworks with open-source tools highlighted.

## Results

### Genetic test use trends in the U.S. health care system

Use of genetic tests can be broadly categorized into diagnostic, predictive, and reproductive applications; however, to meet standards of clinical safety and quality, a genetic test must show sufficient analytical validity, clinical validity, and clinical utility before clinical translation and implementation can occur within regulatory and payer coverage frameworks.^24^

Genetic tests that incorporate multiomic information and the CDSs required for their implementation will still be expected to meet these performance standards, while also demonstrating sufficient economic value relative to costs.^27^ The Centers for Disease Control and Prevention's (CDC) Public Health Genomics and Precision Health Knowledgebase curates various genetic test evaluation frameworks and evidentiary reports for genetic test performance outcomes for a wide range of health conditions, patient populations, and clinical settings.^28,29^

Although the number of genetic tests from CLIA-certified laboratories has increased in the NCBI Genetic Test Registry, these data do not provide an indication of current test utilization and spending within the U.S. health care landscape. An examination of the health care market diffusion patterns for various types of genetic tests and their associated clinical domain applications and challenges would help provide a roadmap for which to begin to prepare for the more technologically and computationally intensive multiomics translational tests.

A recent study of a U.S. commercial payer database spanning 28 health plans, all 50 states, and ∼40 million covered lives reported a total of 75,000 genetic tests, with 14,000 new CLIA-certified laboratory genetic tests (single and multiple genes, exomes, whole genome, microarrays, sequencing, karyotype, and circulating cell-free DNA) coming onto market during 2014–2017, and ∼10 new genetic tests coming onto the market daily.^30^ Claims were curated into clinical domains by current procedural terminology (CPT) code and laboratory identification number to analyze genetic test utilization and spending patterns by quarter for the 3-year study period. Prenatal tests (33–43%), hereditary cancer tests (∼30%), oncology diagnostics and treatment (∼10%), and pharmacogenetic tests (<5%) accounted for the highest percentage of U.S. genetic test spending.

When reimbursed, genetic tests for diagnostics have strong rates and new CPT codes incorporating next-generation sequencing are coming online annually and becoming integrated into electronic medical records and payment systems. In 2018, ∼30 new CPT codes specific to PM (primarily oncology) were added, with CPT code 8146 for exome sequencing analysis with a maximum allowable limit of U.S. $12,000.^31^ However, there are a wide range of incompatibilities between the evidence review process utilized within the insurance industry and the advanced technologies utilized in PM genetic tests. A review of three insurance coverage framework approaches with Medicare's national coverage determination process identified a number of possible evidence review pathways that could be revised to better allow for expanded access to next-generation tumor sequencing genetic tests.^32^ It should be noted that although genetic test use and reimbursement cost trends do not constitute PM directly on their own, they are an indication of the foundational health system tools and architectural components required for PM implementation.

### Workforce development

Mechanisms for ensuring adequate health care provider training in genetics/genomics are widely recognized as including academic and residency programs subject to accreditation requirements, licensure and specialty certification examination proficiency, continuing education licensure requirements, and point-of-care education mechanisms embedded within health care system information technology infrastructure. Table 2 highlights salient health care provider education and training resources by professional domain. Genetics/genomics education competencies and frameworks are available for generalist and specialty medical practice areas, for nongenetic health professionals, and for providers who received education and training before the PM movement.^33–35^

**Table 2.** Selected Genetics/Genomics Education and Training Resources by Health Care Professional Domain

Resource name	Website
Multidisciplinary	
American Society of Human Genetics (health care providers)	https://www.ashg.org/press/healthprofessional.shtml
	https://www.ashg.org/education/
American Society of Health-System Pharmacists	https://www.ashp.org/Pharmacy-Practice/Resource-Centers/Pharmacogenomics
Centers for Disease Control and Prevention	https://www.cdc.gov/genomics/resources/educational.htm
National Library of Medicine, Precision Medicine	https://ghr.nlm.nih.gov/resources#precision-medicine
NHGRI Genomics Competency and Curricular Resources	https://www.genome.gov/For-Health-Professionals/Provider-Genomics-Education-Resources
Method for Introducing a New Competency (MINC)	https://genomicsintegration.net/
Physicians	
American Academy of Family Physicians	https://www.aafp.org/afp/topicModules/viewTopicModule.htm?topicModuleId=56
American Academy of Pediatrics, Council on Genetics	https://pediatrics.aappublications.org/committee_on_genetics
American Board of Medical Genetics and Genomics	http://abmgg.org/
American College of Medical Genetics and Genomics	https://www.acmg.net/
American College of Obstetricians and Gynecologists	https://www.acog.org/About-ACOG/ACOG-Departments/Genetics?IsMobileSet=false
American Medical Association, Precision Medicine	https://www.ama-assn.org/delivering-care/precision-medicine
American Society of Clinical Oncology	https://www.asco.org/practice-policy/cancer-care-initiatives/genetics-toolkit/assessing-managing-your-patients-hereditary
Association of American Medical Colleges	https://www.aamc.org/
Association of Professors of Human and Medical Genetics	https://www.aphmg.org/
Jackson Laboratories Clinical and Continuing Education	https://www.jax.org/education-and-learning/clinical-and-continuing-education#
Inter-Society Coordinating Committee for Practitioner Education in Genomics (ISCC)	https://www.genome.gov/For-Health-Professionals/Inter-Society-Coordinating-Committee-for-Practitioner-Education-in-Genomics
Genetic Counselors	
American Board of Genetic Counseling	https://www.abgc.net/
National Society of Genetic Counselors	https://www.nsgc.org/
Registered Nurses	
American Academy of Nursing, Expert Panel on Genomic Nursing & Health Care	https://www.aannet.org/expert-panels/ep-genomic-nursing--health-care
American Nurses Association	https://www.nursingworld.org/practice-policy/nursing-excellence/ethics/genetics/
Global Genomics Nursing Alliance	https://g2na.org/
International Society of Nurses in Genetics (ISONG)	https://www.isong.org/
National Institute of Nursing Research	https://www.ninr.nih.gov/
Omics Nursing Science and Education Network (ONSEN)	https://omicsnursingnetwork.net/
Oncology Nursing Society, Precision Oncology	https://www.ons.org/learning-libraries/precision-oncology

An adequate supply of health care providers with sufficient education and training to use genetic/genomic information in an ethical and responsible way that respects the dignity, privacy/confidentiality, and welfare of patients and their families, particularly marginalized subpopulations, is an important priority in biomedical technology dissemination. However, the segment of the health care workforce classically trained in genetics/genomics is routinely identified as insufficient for wide scale PM implementation.^36–38^ To ensure adequate diffusion of this valuable specialty into under-resourced areas as the PM movement expands, this could be an area that may benefit from a more focused and structured workforce development analysis initiative, such as the process used by the U.S. Health Resources and Services Administration's (HRSA) National Center for Health Workforce Analysis.^39^

#### Physicians

Despite its recognized importance, number of medical geneticists and medical trainees entering clinical genetics is low, and approximately two-thirds of medical geneticists are approaching retirement age.^40–42^ As of January 2018, the American Board of Medical Genetics and Genomics reported a total of *n*=2937 diplomates (*n*=1583 MD clinical genetics and genomics; *n*=155 PhD medical genetics; *n*=323 clinical biochemical genetics; *n*=775 clinical cytogenetics and genomics; *n*=743 clinical molecular genetics and genomics, and *n*=49 clinical biochemical/molecular genetics; *n*=63 medical biochemical genetics; *n*=9 molecular genetic pathology).^43^ To increase physician trainees entering this specialty, a number of career development strategies and educational framework mechanisms are proposed, with a particular emphasis on development of premedical undergraduate genomics curriculums.^37,40^

#### Genetic counselors

According to the American Board of Genetic Counseling, there are presently ∼5172 certified genetics counselors in the United States who practice in cancer, prenatal, and pediatric settings located in university medical settings, public/private hospital facilities, and diagnostic laboratories.^44,45^ Presently in the United States, there are discrepant reports as to the presence and severity of a certified genetic counselor (CGC) shortage. A 2016 industry-sponsored workforce study of U.S.-based CGCs for 2017–2026 found that the U.S. CGC workforce had grown by 88% from 2006 to 2016, and is projected to expand another 72% in the next 10 years.^46^ Conflicting with these trends are peer-reviewed scientific reports that identify insufficient CGCs across various specialties and inadequate distribution across geographic locales, particularly rural settings and the southern United States.^47^ To address gaps in coverage, alternative health care delivery models to increase genetic counseling access in CGC-desert areas include telegenetic medicine and interactive web consults, greater health care workforce provider education and training (i.e., registered nurses [RNs], licensed clinical social workers) in CGC-limited settings, group counseling sessions, and cultivation of innovative institutional collaborations between rural facilities and larger academic partners.^48–50^

#### Nurses

RNs are the largest body of health care provider and play a key role in the provision of health care services to patients, families, and communities. The U.S. nursing workforce consists of 4,015,250 RNs (2,857,180 directly employed within the United States).^51^ However, there are approximately *n*=350 nurse clinician, educator, and scientist members in the professional advocacy organization for genetics/genomics and approximately *n*=425 graduates of the NIH National Institute of Nursing Research's (NINR) Summer Genetics Institute.^51–53^ To facilitate content translation to the broader nursing community, essential genetic and genomic competencies, curricula guidelines, and outcome indicators were developed for U.S. entry level and advanced practice nurse programs and were endorsed and implemented by numerous professional nursing accreditation and certification organizations.^54,55^ Knowledge standards for omic science doctoral nursing research programs were recently developed, in addition to genetic and genomic competencies for nursing informatics.^56,57^ Lastly, a new Global Genomics Nursing Alliance (G2NA) aims to increase the integration of genomics into routine patient care in the general nursing community by providing a global catalogue of nursing education competencies and genomic nursing practice resources.^58^

### Health system implementation frameworks and CDS

A number of learning health systems are pioneering PM advances through the implementation of genomic medicine screening programs to prevent, detect, and treat adverse health outcomes and chronic diseases. Examples of pharmacogenomic and genomic medicine implementation pilots integrated into health care delivery systems include: North Shore University Health System “DNA10K” population health primary care initiative, Intermountain Healthcare precision oncology program, and Geisinger MyCode Community Health Initiative.^8,59–66^

Although the American College of Medical Genetics and Genomics has issued a policy statement advocating for the return of information on 59 medically actionable genes after clinical exome and whole genome sequencing, complexities of incorporating genomic information into routine clinical practice are formidable in the absence of specialized training.^67^

Clinical implementation barriers for genetic tests and technologies are widely cited and contribute considerably to variable health care system uptake, even in the presence of documented clinical effectiveness.^68^ The NHGRI Implementing GeNomics In pracTicE (IGNITE) Network framework identifies best practices and lessons learned to achieve sustainable adoption of genomic technologies in health care systems.^69–71^

Key components of best-practice implementation within health systems are the structured use of genomic information in clinical care that is linked to (and ideally) supported within the EHR to be guided by CDS. CDS “provides clinicians, staff, patients or other individuals with knowledge and person-specific information, intelligently filtered or presented at appropriate times, to enhance health and healthcare.”^72^

CDS comprises a variety of rules, resources, and interventions that support clinical workflows at the point of health care delivery by a variety of health care providers, including computerized alerts and reminders to practitioners and patients, evidenced-based clinical guidelines, condition or test result-specific order sets, patient data reports and summaries, documentation templates, diagnostic support information, and contextual references to provide accurate health decision making.^73^

There are three levels of CDS, including passive (nonmandatory EHR resources available to a practitioner at the time of health care), asynchronous (provides information to a practitioner outside of clinical workflow such as an inbox message at the start of a workday), and active (presents information needed for decision making during the practitioner's clinical workflow, “just in time”).^65^ There are a wide range of guides and open source tools for genetic test implementation, including but not limited to: NHGRI IGNITE program's Genomic Medicine Knowledge Base, the Pharmacogenomics Clinical Annotation Tool (PharmCAT), the Displaying and Integrating Genetic Information through the EHR (DIGITizE) program, and the CDS knowledge base (CDS-KB).^74,75^ For example, the IGNITE SPARK Toolbox feature provides a range of institutional assessment and CDS tools for genetic test implementation within health care systems, such as the CYP2C19 pharmacogenetic test and associated clopidogrel dosing procedure. Given 404 currently FDA approved pharmacogenomic biomarkers used in drug labeling, CDS tools are and will become essential to connecting patient genetic test results to the appropriate pharmacogenomic drug label indication.^3^

A key consideration to the implementation of any genetic test, PM CDS initiative for nongenetics health care providers, is ensuring that CDS alerts are structured into clinical workflows in ways that are not excessively burdensome.^75^ Consequences of poorly implemented CDS are that providers minimize and ignore safety alerts thus jeopardizing patient safety in the event of a true alert that is inappropriately ignored or silenced.^76,78^ Clinician burnout is identified as a key health policy issue requiring immediate action by a number of federal, regional, public, and private stakeholders to preserve well-being of practitioners in addition to providing high-quality and safe clinical care.^79^

There are broader patient safety frameworks available to guide judicious implementation of CDS so that its use achieves the aim of delivering safe and effective clinical interventions optimized for clinician and health care system workflows. Examples include the Health Information Technology (IT) Safety resource from The U.S. Department of Health and Human Services' Office of the National Coordinator for Health Information Technology (ONC), and the Agency for Healthcare Research and Quality Alert Fatigue resource.^73,80^

## Discussion

Health care delivery institutions and systems are beginning to implement genetic tests that are driving PM. Genetic test use is trending up in terms of rapid U.S. market expansion as evidenced by increased annual CPT claim registry and utilization outcomes in the commercial sector, particularly for prenatal and oncology applications, resulting in improved diagnostic and therapeutic tools for a wide range of hereditary and complex conditions. However, ELSI of genetic/genomic test implementation continues to be a priority to ensure adequate access and use of these cutting-edge biomedical advances, particularly for marginalized and vulnerable populations and ethnic minorities.

To ensure safe adoption and clinical translation of PM, health care systems have an ethical responsibility to ensure that their providers and front-line staff are prepared to order, use, and interpret genetic test information. In addition to accommodating genomic information use in CDS and EHR infrastructures, health care systems need to pay particular attention to nongeneticist health care provider workforce adequacy and preparedness to ensure safe application of these advances and drive effective workflow implementation.

There are a number of high-quality evidenced-based educational resources and CDS tools available. CDS and point-of-care “just-in-time” alerts and rapid education intervention tools are a primary pathway for health care providers to use and implement PM, but they must be wisely implemented to avoid health care provider alert fatigue and clinician burnout. This field can be particularly hampered by health care system EHR infrastructure limitations that are unable to support CDS innovations and workflow redesigns involving input and feedback from front-line providers. However, health care systems with cultures fostering strong partnerships between administrative leaders and front-line providers and staff, coupled with reasonable goal setting, will help facilitate PM translation interests.

## Conclusion

The (PM) era presents an exciting yet daunting challenge to implement targeted molecular diagnostic and therapeutic treatments using genetic/genomic and omic information into routine health care settings. The fruits of large scale genomic and biobank research initiatives, population health programs, and the proliferation of bioinformatic tools provide learning health systems and front-line health providers powerful resources to prevent and treat a wide range of hereditary and chronic conditions across a wide range of patient care settings. Strategic planning to support health system infrastructure development that is able to incorporate genetic/genomic information, efficient EHR documentation, CDS capacity, and an adequate number of health care providers trained to use genetic/genomic information will ensure ethical and safe adoption of PM clinical translation interests.

## Abbreviations Used

CDS: clinical decision support
CGC: certified genetic counselor
CLIA: Clinical Laboratory Improvement Amendments
CPT: current procedural terminology
EHR: electronic health record
ELSI: ethical, legal, and social implications
eMERGE: electronic Medical Records and Genomics
FDA: Food and Drug Administration
IGNITE: Implementing GeNomics In pracTicE
NCBI: National Center for Biotechnology Information
NHGRI: National Human Genome Research Institute
NIH: National Institutes of Health
NINR: National Institute of Nursing Research
PM: precision medicine
RN: registered nurse

## References

[1] Collins FS, Varmus H A new initiative on precision medicine. N Engl J Med. 2015;372:793–795 25635347 10.1056/NEJMp1500523 PMC5101938

[2] National Center for Biotechnology Information. Genetic testing registry. www.ncbi.nlm.nih.gov/gtr Accessed 229, 2020

[3] Food and Drug Administration. Table of pharmacogenomic biomarkers in drug labeling. Science and research, drugs. www.fda.gov/drugs/science-and-research-drugs/table-pharmacogenomic-biomarkers-drug-labeling Accessed 229, 2020

[4] The All of Us Research Program Investigators. The “All of Us” research program. N Engl J Med. 2019;381:668–676 31412182 10.1056/NEJMsr1809937 PMC8291101

[5] NHGRI. eMERGE network.www.genome.gov/Funded-Programs-Projects/Electronic-Medical-Records-and-Genomics-Network-eMERGE Accessed 229, 2020

[6] Sudlow C, Gallacher J, Allen N, et al. UK biobank: an open access resource for identifying the causes of a wide range of complex diseases of middle and old age. PLoS Med. 2015;12:1–10 10.1371/journal.pmed.1001779 PMC4380465 25826379

[7] National Academies of Sciences, Engineering, and Medicine. Implementing and evaluating genomic screening programs in health care systems: Proceedings of a Workshop. National Academies Press, Washington, DC 2018 29787046

[8] LevitL, Kim E, McAneny B, et al. Implementing precision medicine in community-based oncology programs: three models. J Onc Prac. 2019;15:325–329 10.1200/JOP.18.00661 30802151

[9] Auffray C Interview with a thought leader on systems medicine—Charles Auffray, PhD. Syst Med. 2018;1:11–12

[10] Auffray C, Balling R, Barroso I, et al. Making sense of big data in health research: towards and EU action plan. Genome Me. 2016;8:1–13 10.1186/s13073-016-0323-y PMC4919856 27338147

[11] Chan S Loscalzo J. The emerging paradigm of network medicine in the study of human disease. Circ Res. 2012;111:359–374 10.1161/CIRCRESAHA.111.258541 PMC3425394 22821909

[12] El-Manzalawy Y, Hsieh T, Shivakumar M, et al. Min-redundancy and max-relevance multi-view feature selection for predicting ovarian cancer survival using multi-omics data. BMC Med Genomics. 2018;11(Suppl 3):71; 21–31. 30255801 10.1186/s12920-018-0388-0 PMC6157248

[13] Schussler-Fiorenza Rose S, Contrepois K, Moneghetti KJ, et al. A longitudinal big data approach for precision health. Nat Med. 2019;25:792–804 31068711 10.1038/s41591-019-0414-6 PMC6713274

[14] Clark D, Dhanasekaran S, Petralia F, et al. Integrated proteogenomic characterization of clear cell renal cell carcinoma. Cell. 2019; 179:964–983 31675502 10.1016/j.cell.2019.10.007 PMC7331093

[15] Tang HHF, Sly PD, Holt PG, et al. Systems biology and big data in asthma and allergy: recent discoveries and emerging challenges. Eur Respir J 2020;55:1900844 31619470 10.1183/13993003.00844-2019

[16] NHGRI. Genomics and medicine. www.genome.gov/health/Genomics-and-Medicine Accessed 229, 2020

[17] Harvard University Catalyst. Network medicine. A course series on science in biology and medicine. https://catalyst.harvard.edu/services/networkmedicine Accessed 229, 2020

[18] NHGRI. Talking glossary of genetic terms. Personalized medicine. www.genome.gov/genetics-glossary/Personalized-Medicine Accessed 229, 2020

[19] NCI. NCI dictionary of cancer terms. Precision medicine. www.cancer.gov/publications/dictionaries/cancer-terms/def/precision-medicine Accessed 229, 2020

[20] Georgetown University Medical Center. First international conference in systems and network medicine: Application of systems science and thinking to biomedicine. Systems medicine definition. https://sites.google.com/georgetown.edu/sysmedconf/home Accessed 229, 2020

[21] CDC. Precision health: Improving health for each of us and all of us. www.cdc.gov/genomics/about/precision_med.htm Accessed 229, 2020

[22] Khoury MJ, Galea S Will precision medicine improve population health? JAMA. 2016;316:1357–1358 27541310 10.1001/jama.2016.12260 PMC6359904

[23] Xu J, Yang P, Xue S, et al. Translating cancer genomics into precision medicine with artificial intelligence: applications, challenges, and future perspectives. Hum Genet. 2019;W138:109–124 10.1007/s00439-019-01970-5 PMC6373233 30671672

[24] National Academies of Sciences, Engineering, and Medicine. An Evidence Framework for Genetic Testing. Health and Medicine Division, Board on Healthcare Services, Board on the Health of Select Populations; Committee on the Evidence Base for Genetic Testing. National Academies Press, Washington, DC 2017

[25] Paker L, Sankar P, Boyer J, et al. Normative and conceptual ELSI research: what it is, and why it is important. Gen Med. 2019;21:505–509 10.1038/s41436-018-0065-x 29970926

[26] NHGRI. ELSI research domains. www.genome.gov/Funded-Programs-Projects/ELSI-Research-Program/domains Accessed 229, 2020

[27] Ginsburg G, Phillips K Precision medicine: from science to value. Health Aff. 2018;37:694–701 10.1377/hlthaff.2017.1624 PMC5989714 29733705

[28] CDC. Public health genomics and precision health knowledge base (v6.0). https://phgkb.cdc.gov/PHGKB/phgHome.action?action=home Accessed 229, 2020

[29] Parkinson D, McCormack R, Keating S, et al. Evidence of clinical utility: an unmet need in molecular diagnostics for patients with cancer. Clin Cancer Res. 2014;20:1428–1444 24634466 10.1158/1078-0432.CCR-13-2961

[30] Phillips K, Deverka P, Hooker G, et al. Genetic test availability and spending: where are we now? Where are we going? Health Aff. 2018;37:710–716 10.1377/hlthaff.2017.1427 PMC5987210 29733704

[31] Cheong Q, Chilukuri S, Quigley D, et al. Genetic Testing: Opportunities to Unlock Value in Precision Medicine. Pharmaceuticals & Medical Products. 2018 McKinsey & Company: 1–7. www.mckinsey.com/industries/pharmaceuticals-and-medical-products/our-insights/genetic-testing-opportunities-to-unlock-value-in-precision-medicine Accessed 229, 2020

[32] Trosman J, Weldon C, Gradishar W, et al. From the past to the present: insurer coverage frameworks for next-generation tumor sequencing. Value Health. 2018:21;1062–1068 30224110 10.1016/j.jval.2018.06.011 PMC6374027

[33] Korf B, Berry A, Limson M, et al. Framework for development of physician competencies in genomic medicine: report of the competencies working group of the Inter-Society Coordinating Committee for physician education in genomics. Gen Med. 2014;16:804–809 10.1038/gim.2014.35 24763287

[34] Talwar D, Tseng T, Foster M, et al. Genetics/genomics education for nongenetic health professionals: a systematic review. Gen Med. 2017;19:725–732 10.1038/gim.2016.156 27763635

[35] Reed E, Taber L, Nissen T, et al. What works in genomics education: outcomes of an evidenced-based instructional model for community-based physicians. Gen Med. 2016;18:737–745 10.1038/gim.2015.144 26583682

[36] Calzone K, Jenkins J, Culp S, et al. Hospital nursing leadership led interventions increased genomic awareness and education intent in Magnet settings. Nurs Outlook. 2018;66:244–253 29544651 10.1016/j.outlook.2017.10.010 PMC5949252

[37] Hyland K, Garber K, Dasgupta S From helices to health: undergraduate medical education in genetics and genomics. Pers Med. 2019;16:211–220 10.2217/pme-2018-0081 30489214

[38] Pan V, Rashar B, Pothast R, et al. Expanding the genetic counseling workforce: program directors' views on increasing the size of genetic counseling graduate programs. Gen Med. 2016;18:842–849 10.1038/gim.2015.179 26741410

[39] Health Resources and Services Administration (HRSA). State Level Projections of Supply and Demand for Behavioral Health Occupations: 2016–2030. Technical documentation for HRSA health workforce simulation model. Bureau of Health Workforce National Center for Health Workforce Analysis. Rockville, MD: DHHS. 2018

[40] Cichon M, Feldman G Opportunities to improve recruitment into medical genetics residency programs: survey results of program directors and medical genetics residents. Gen Med. 2014;16:413–418 10.1038/gim.2013.161 24136619

[41] Ferreira C, Regier D, Hadley D, et al. Medical genetics and genomic medicine in the USA. Part 1: history, demographics, legislation and burden of disease. Mol Gene Genomic Med. 2017;5:307–316 10.1002/mgg3.318 PMC5511798 28717657

[42] Regier D, Ferreira C, Hart S, et al. Medical genetics and genomic medicine in the USA. Part 2: reproductive genetics, newborn screening, genetic counseling, training, and registries. Mol Genet Genomic Med. 2017;5:621–630 29178644 10.1002/mgg3.343 PMC5702569

[43] American Board of Medical Genetics and Genomics. Number of ABMGG certified specialists in genetics and genomics by examination year. http://www.abmgg.org/pdf/Statistics%20for%20Webpage.pdf Accessed 229, 2020

[44] American Board of Genetic Counseling, Inc. ABGC is now 5000 strong and growing. www.abgc.net/for-diplomates Accessed 229, 2020

[45] National Society of Genetic Counselors. Genetic counselor workforce initiatives. Does a genetic counselor workforce shortage exist. www.nsgc.org/page/genetic-counselor-workforce-initiatives-532 Accessed 229, 2020

[46] The Genetic Counselor Workforce Working Group. Workforce study executive study. 2016 www.abgc.net/research-resources/latest-research Accessed 229, 2020

[47] Villegas C, Haga S Access to genetic counselors in the southern United States. J Pers Med. 2019;9:1–12 10.3390/jpm9030033 PMC6789777 31266141

[48] Boothe E, Kaplan J Using telemedicine in Mississippi to improve patient access to genetic services. J Gen Serv. 2018;27:320–322 10.1007/s10897-017-0192-6 29264692

[49] Cohen S, Huziak R, Gustafson S Analysis of advantages, limitations, and barriers of genetic counseling service delivery models. J Gen Counsel. 2016;25:1010–1018 10.1007/s10897-016-9932-2 26762366

[50] Gammon B, Otto L, Wick M, et al. Implementing group prenatal counseling for expanded noninvasive screening options. J Gen Counsel. 2018;27:894–901 10.1007/s10897-017-0178-4 29247311

[51] National Council for State Boards of Nursing. Progress and precision: the NCSBN environmental scan. J Nur Reg. 2018;8:S3–S6

[52] Hickey K, Taylor J, Barr T, et al. Nursing genetics and genomics: the international society of nurses in genetics (ISONG) survey. Nur Educ Tod. 2018;63:12–17 10.1016/j.nedt.2018.01.002 PMC6461386 29407254

[53] NINR. Training at NINR (NIH campus). Summer genetics institute. www.ninr.nih.gov/training/trainingopportunitiesintramural/summergeneticsinstitute Accessed 229, 2020

[54] Calzone K, Jenkins J, Prows C, et al. Establishing the outcome indicators for the essential for the essential nursing competencies and curricular guidelines for genetics and genomics. J Prof Nur. 2011;27:179–191 10.1016/j.profnurs.2011.01.001 PMC3099038 21596359

[55] Greco K, Tinley S, Seibert D Development of the essential genetic and genomics competencies for nurses with graduate degrees. Ann Rev Nur Res. 2011;29:173–190 10.1891/0739-6686.29.173 22891504

[56] McCormick K, Calzone K Genetic and genomic competencies for nursing informatics internationally. Stud Health Technol Inform. 2017;232:152–164 28106593 PMC10411530

[57] Regan M, Engler M, Coleman C, et al. Establishing the genomic knowledge matrix for nursing science. J Nur Schol. 2019;51:50–57 10.1111/jnu.12427 PMC6329656 30272391

[58] Calzone K, Kirk M, Tonkin E Increasing nursing capacity in genomics: overview of existing global genomics resources. Nur Educ Today. 2018;69:53–59 10.1016/j.nedt.2018.06.032 PMC6112149 30007148

[59] North Shore University Health System. NorthShore and color unlock the power of genomics in routine care. www.northshore.org/newsroom/press-releases/northshore-and-color-unlock-the-power-of-genomics-in-routine-care Accessed 229, 2020

[60] Lemke A, Thompson J, Hulick PJ, et al. Primary care physician experiences utilizing a family health history tool with electronic health-record integrated clinical decision support: an implementation process assessment. J Community Genet. 2020:1–12. DOI: 10.1007/s12687-020-00454-8 32020508 PMC7295926

[61] Manickam M, Buchanan A, Schwartz M, et al. Exome sequencing-based screening for BRCA 1/2 expected pathogenic variants among adult biobank participants. JAMA Open. 2018;1:1–14 10.1001/jamanetworkopen.2018.2140 PMC6324494 30646163

[62] National Academies of Sciences, Engineering, and Medicine. Genomics-Enabled Learning Health Care Systems: Gathering and Using Genomic Information to Improve Patient Care and Research. Workshop summary. National Academies Press, Washington, DC 2015 25996024

[63] Nadauld L, Ford J, Pritchard D Strategies for clinical implementation: precision oncology at three distinct institutions. Health Aff. 2018;37:751–756 10.1377/hlthaff.2017.1575 29733728

[64] Poonen P, Duffy J, Hintze B, et al. Genomic analysis of metastatic solid tumors in veterans: findings from the VHA national precision oncology program. J Clin Oncol. 2019;37 (suppl):1–13 32914016 10.1200/PO.19.00075 PMC7446382

[65] Williams M Early lessons from the implementation of genomic medicine programs. Ann Rev Gen Hum Gen. 2019;20:389–411 10.1146/annurev-genom-083118-014924 30811224

[66] Williams W, Buchanan A, Davis F, et al. Patient-centered precision health in a learning health care system: Geisinger's genomic medicine experience. Health Aff. 2018;37:757–764 10.1377/hlthaff.2017.1557 29733722

[67] Kalia S, Adelman K, Bale SJ, et al. Recommendations for reporting of secondary findings in clinical exome and genome sequencing, 2016 update (ACMG SF v2.0): a policy statement of the American College of Medical Genetics and Genomics. Gen Med. 2017;19:249–255 10.1038/gim.2016.190 27854360

[68] Zebrowski A, Ellis D, Barg F, et al. Qualitative study of system level factors related to genomic implementation. Gen Med. 2019;21:1534–1540 10.1038/s41436-018-0378-9 PMC6533158 30467402

[69] Cavallari L, Beitelshees A, Blake K, et al. The IGNITE pharmacogenetics working group: an opportunity for building evidence with pharmacogenetic implementation in a real-world setting. Clin Trans Sci. 2017;10:143–146 10.1111/cts.12456 PMC5421730 28294551

[70] Levy K, Blake K, Fletcher-Hoppe C, et al. Opportunities to implement a sustainable genomic medicine program: lessons learned from the IGNITE network. Gen Med. 2019;21:743–747 10.1038/s41436-018-0080-y PMC6330142 29997387

[71] Sperber N, Carpenter J, Cavallari L, et al. Challenges and strategies for implementing genomic services in diverse settings: experiences from the Implementing GeNomics In practice (IGNITE) network. BMC Med Gen. 2017;10: 1–11 10.1186/s12920-017-0273-2 PMC5441047 28532511

[72] Office of the National Coordinator for Health Information Technology. (2019). Health IT safety. What is clinical decision support. www.healthit.gov/topic/safety/clinical-decision-support Accessed 229, 2020

[73] Office of the National Coordinator for Health Information Technology. (2019). Health IT safety. www.healthit.gov/topic/health-it-safety Accessed 229, 2020

[74] IGNITE. Genomic medicine knowledge base (GMKB). The knowledge hub for genomic medicine. https://gmkb.org Accessed 229, 2020

[75] Volpi S, Bult C, Chisholm R, et al. Research directions in the clinical implementation of pharmacogenomics—An overview of US programs and projects. Clin Pharm Ther. 2018;103:778–786 10.1002/cpt.1048 PMC5902434 29460415

[76] Ancker J, Edwards A, Nosal S, et al. Effects of workload, work complexity, and repeated alerts on alert fatigue in a clinical decision support system. BMC Med Inf Dec Mak. 2017;17:1–9 10.1186/s12911-017-0430-8 PMC5387195 28395667

[77] Baysari M, Zheng W, Li L, et al. Optimising CDS to transform medication safety and reduce prescriber burden: study protocol for a mixed-methods evaluation of drug-drug interaction alerts. BMJ Open. 2019;9:1–7 10.1136/bmjopen-2018-026034 PMC6701635 31427312

[78] ECRI Institute. Top 10 patient safety concerns for health care organizations in 2017. www.ecri.org/EmailResources/PSRQ/Top10_2017/HRC_Apr17Reporter_Top10_final.pdf Accessed 229, 2020

[79] National Academies of Sciences, Engineering, and Medicine. Taking Action Against Clinician Burnout: A Systems Approach to Professional Wellbeing. Washington, DC: National Academies Press 2019 31940160

[80] Agency for Healthcare Research and Quality. Patient safety primer. Alert fatigue. https://psnet.ahrq.gov/primer/alert-fatigue Accessed 229, 2020

